# Tesmin, Metallothionein-Like 5, is Required for Spermatogenesis in Mice^†^

**DOI:** 10.1093/biolre/ioaa002

**Published:** 2020-01-09

**Authors:** Asami Oji, Ayako Isotani, Yoshitaka Fujihara, Julio M Castaneda, Seiya Oura, Masahito Ikawa

**Affiliations:** 1 Research Institute for Microbial Diseases, Osaka University, Suita, Osaka, Japan; 2 Graduate School of Pharmaceutical Sciences, Osaka University, Suita, Osaka, Japan; 3 Immunology Frontier Research Center, Osaka University, Suita, Osaka, Japan; 4 The Institute of Medical Science, The University of Tokyo, Tokyo, Japan

**Keywords:** spermatogenesis, meiotic arrest, male infertility, knockout

## Abstract

In mammals, more than 2000 genes are specifically or abundantly expressed in testis, but gene knockout studies revealed several are not individually essential for male fertility. *Tesmin* (*Metallothionein-like 5*; *Mtl5*) was originally reported as a testis-specific transcript that encodes a member of the cysteine-rich motif containing metallothionein family. Later studies showed that *Tesmin* has two splicing variants and both are specifically expressed in male and female germ cells. Herein, we clarified that the long (*Tesmin-L*) and short (*Tesmin-S*) transcript forms start expressing from spermatogonia and the spermatocyte stage, respectively, in testis. Furthermore, while *Tesmin*-deficient female mice are fertile, male mice are infertile due to arrested spermatogenesis at the pachytene stage. We were able to rescue the infertility with a *Tesmin-L* transgene, where we concluded that TESMIN-L is critical for meiotic completion in spermatogenesis and indispensable for male fertility.

## Teaser

TESMIN protein, a member of metallothionein family, is essential for mouse spermatogenesis, especially in the meiotic stage.

## Introduction

Spermatogenesis is a complicated process involving mitotically dividing spermatogonial cells, spermatocytes dividing meiotically, and spermatid maturation into spermatozoa. While spermatogonial stem cells reside at the basement of the seminiferous tubules and continuously produce progeny cells to maintain the stem cell pool, some of the progeny cells start differentiation into primary spermatocyte (4C). The primary spermatocytes undergo meiosis I to form secondary spermatocytes (2C) and meiosis II to form spermatids (1C). The spermatids elongate their cilia and drastically change their morphology to form mature spermatozoa, which are released into the lumen of seminiferous tubules. Each of these events is supported by stage- and cell-specific gene expression.

In mammals, gene-expression analysis estimates that more than 2300 genes are expressed predominantly in the male germ line [1], and these testis-enriched genes are believed to play critical roles for male fertility. While knockout (KO) mouse studies have revealed essential genes for male fertility, recent CRISPR/Cas9-mediated KO studies suggested that many of the testis-enriched genes are not individually essential [2]. One can posit that paralogous genes may complement the testis-specific gene functions. However, it should be noted that many genes shown essential for male fertility are simultaneously expressed with their paralogues during spermatogenesis (e.g., *Ccna2/Ccna1* [3], *Hspa1a/Hspa2* [4]). Thus, the KO approach remains suitable to determine whether the target gene has an essential role in vivo.

Metallothioneins (MTs) belong to a group of low-molecular-weight cysteine-rich proteins, which bind to heavy metal ions via their cysteine-rich (CXC) motif [5, 6]. MTs fall into four subgroups (MT1-4), *Mt1* and *Mt2* are ubiquitously expressed in mice, *Mt3* is expressed mostly in the brain, and *Mt4* was detected in stratified squamous epithelial cells. All are located on a single chromosome (chromosome 8 in mice and chromosome 11 in human) [7]. Additionally, *Tesmin* was identified as a fifth member, which is strongly expressed in testis [8]. It was discovered that *Tesmin* encodes two variants with the longer variant having an additional 180 amino acids at the N-terminus [9]. We refer to the long form as TESMIN-L and the short form as TESMIN-S, respectively. Both TESMIN-variant proteins contain the CXC-domain like cysteine-rich domains and a hinge region as described in previous reports [9, 10]. Since TESMIN-L had been reported to change its localization in response to metal stress in vivo and in vitro [9, 11], TESMIN proteins are assumed to chelate metal ions like other MTs. However, TESMIN-L and TESMIN-S have some differences compared with other members of the MT family. For example, TESMIN proteins are relatively larger than other family members: TESMIN-L consists of 475 amino acids and TESMIN-S consists of 295 amino acids, while MT1–4 consists of 61, 61, 68, and 62 amino acids, respectively. Moreover, *Tesmin* is located on chromosome 19 in mice, while the other *Mt1–4* genes cluster on chromosome 8.

TESMIN-L has also been reported to translocate from the cytoplasm to the nucleus before meiotic division even without metal ion stress [9, 11]. Furthermore, as described in a previous study, meiosis-stage-specific expression of *Tesmin* suggests a germ-cell-specific function independent of the metal ion stress response [12]. Despite these findings, it remains unclear whether TESMIN proteins play critical roles in meiosis and which isoform (TESMIN-L and TESMIN-S) works in testis. In the present study, we generated *Tesmin* KO mice and demonstrated its essential role in the completion of meiosis in male germ cells.

## Materials and methods

### Animals

All animal experiments were conducted in accordance with the guidelines of “Animal experiment rules” established by the Research Institute for Microbial Diseases, Osaka University, and were approved by the Animal Care and Use Committee of the Research Institute for Microbial Diseases, Osaka University. B6D2F1 and ICR mice were purchased from CLEA (Tokyo, Japan) and SLC (Shizuoka, Japan).

### RT-PCR

Mouse cDNA was prepared from various tissues of adult ICR, and testes from 0 to 35 days after birth. RT-PCR was performed using 10 ng of cDNA with the following forward and reverse primers: 5′-CCAGCAGGGCTAGGGATAGA-3′ and 5′-CATGGGGCTGGAGTCTTTCTTCC-3′ for *Tesmin-S*, 5′-ATGGAGGACGCGCTGCTCGG-3′ and 5′-CATGGGGCTGGAGTCTTTCTTCC-3′ for *Tesmin-L*, 5′-AAGTGTGACGTTGACATCCG-3′ and 5′-GATCCACATCTGCTGGAAGG-3′ for the *Actb* gene. The amplification conditions were 1 min at 94 °C, followed by 40 cycles (for *Tesmin-S*) or 35 cycles (for *Tesmin-L*, *Actb*) of 94 °C for 30 s, 65 °C for 30 s, and 72 °C for 30 s, with a final 1-min extension at 72 °C.

### Plasmid construction

The cDNA encoding the mouse *Flag-Tesmin-S* and *Flag-Tesmin-L* variants were amplified by PCR using wild-type (WT) testis cDNA as a template. The PCR primer sets used were 5′-AAGAATTCGCCGCCATGGACTACAAAGACGATGACGACAAGGGCGGCGTGATTTGTCAGCTGAAAGG-3′ and 5′-AAGTCGACCTACTCAATTTTCAGCCCCTTGGACTTG-3′ for *Flag-Tesmin-S*, and 5′-AAGAATTCGCCGCCATGGACTACAAAGACGATGACGACAAGGGCGGCGAGGACGCGCTGCTCGG-3′ and 5′-AAGTCGACCTACTCAATTTTCAGCCCCTTGGACTTG-3′ for *Flag-Tesmin-L*. The *Eco*RI and *Sal*I sites included in the PCR primers were used to introduce the amplified cDNA into a pCAG ubiquitous expression vector carrying chicken *Actb* promoter with a cytomegalovirus enhancer [13, 14]. We named them pCAG-Flag-Tesmin-S and pCAG-Flag-Tesmin-L, respectively.

### Antibodies

Rabbit anti-mouse TESMIN-L polyclonal antiserum was produced by immunization with mouse TESMIN-L polypeptide (AYLGATEPGEPLLRALS) (Sigma-Aldrich, MO, USA, IB 1:200). Anti-FLAG antibody (M2, #F1804) from Sigma-Aldrich, anti-CALNEXIN antibody (#sc-11397, IB 1:1000) from Santa Cruz Biotechnology (Santa Cruz, CA, USA), anti-SYCP polyclonal antibody (#ab15092) from Abcam (Cambridge, UK), anti-phospho-histone H2A.X (Ser139) antibody (#05-636) from Merck Millipore (Darmstadt, Germany) were used. Secondary polyclonal antibodies were used as follows: rabbit anti-mouse HRP 1:1000 (Dako, Hamburg, Germany), goat anti-rabbit HRP 1:2000 (Dako).

### Cell culture and transfection

Cos7 cells were maintained with Dulbeccos modified Eagles medium, 100 U/ml of penicillin, 0.1 mg/ml of streptomycin sulfate, and 10% fetal bovine serum under 5% CO_2_ at 37 °C. For transfection, the cells were transfected at approximately 70% confluence with 1 μg of pCAG-Flag-Tesmin-S or pCAG-Flag-Tesmin-L using Lipofectamine LTX & PLUS technology (Life Technologies, Carlsbad, CA, USA) according to the manufacturer’s instructions.

### Immunofluorescence staining of Cos7

Transfected Cos7 cells were seeded onto coverslips in 6-well plates. After 2 days, cells were fixed with 2% paraformaldehyde in phosphate-buffered saline (PBS) for 10 min. After washes with PBS, cells were permeabilized with 0.2% Triton X-100 for 7 min and then blocked with 10% goat serum in PBS. Then, coverslips were incubated with anti-FLAG (1:200) antibody for overnight at 4 °C. After incubation with Alexa Fluor 488 conjugated secondary antibody (1:200) (Thermo Fisher Scientific, MA, USA) at room temperature for 1 h, cells were counterstained with Hoechst33342 (Life Technologies) and mounted on slides.

### Guide RNA design and cloning

*Tesmin*-specific guide sequences (sgRNAs) were designed and inserted into the pX330 plasmid (#42230, Addgene, Cambridge, MA, USA). Validation of DNA cleavage activity of these plasmids was performed as described previously [15, 16].

### Generation of *Tesmin* KO mice and transgenic mice

B6D2F1 superovulated females were mated with B6D2F1 males and then fertilized eggs were collected. Circular pX330 plasmids were injected into one of the pronuclei at 5 ng/μl. Surviving zygotes were cultured in potassium simplex optimization medium (KSOM) medium for 1 day. Developing two-cell embryos were transferred into the oviducts of pseudo-pregnant foster mice. KO mice were genotyped by PCR with the primers 5′-CTCAGAGGTAGGAAATTGAG-3′ and 5′-CTCAAGCGCTCATTGACCTG-3′. The *Tesmin-L* transgenic mouse line was established by pronuclear injection of the CAG-*Tesmin-L* transgene. The cDNA encoding the mouse *Tesmin-L* gene was amplified by PCR with the primers 5′-AAGAATTCGCCGCCATGGAGGACGCGCTGCTCGG-3′ and 5′-AAGTCGACCTACTCAATTTTCAGCCCCTTGGACTTG-3′ using WT testis cDNA as a template. The *Eco*RI and *Sal*I sites included in the PCR primers were used to introduce the amplified *Tesmin-L* cDNA into a pCAG expression vector described above. The transgene fragment (3.7 kb) was digested with *Cla*I, *Pac*I, and *Sca*I and purified by gel electrophoresis. Transgenic mice were genotyped by PCR with primers 5′-GCCTTCTTCTTTTTCCTACAGC-3′ and 5′-AGCAGGTCCAGAGGGAAAGGCAGC-3′. Frozen spermatozoa from *Tesmin* disrupted males (B6D2-*Mtl5*<em1Osb>, RBRC#09987, CARD#2542) and *Tesmin-L* transgenic males (B6D2-*Mtl5*<em1Osb> Tg(CAG-*Mtl5*)1Osb, RBRC#10131, CARD#2604) will be available through RIKEN BRC (http://en.brc.riken.jp/index.shtml) and CARD R-BASE (http://cardb.cc.kumamoto-u.ac.jp/transgenic/).

### Assessment of the fertility of TESMIN deficient mice

Sexually mature male mice of genotypes *Tesmin* +/em1 and KO were caged with B6D2F1 female mice (at least 2 months old) for 3 months, and the number of pups in each cage was counted within a week of birth.

### Immunoblotting

Protein extracts from whole testis were subjected to 10% polyacrylamide gel electrophoresis and blotted onto a polyvinylidene fluoride membrane. Membranes were blocked in 10% skim milk powder in tris-buffered saline with Tween20 and probed with primary (overnight at 4 °C) and secondary (1 h at RT) antibodies in blocking solution. Chemiluminescent signals were detected using ImageQuant LAS 4000 mini (GE Healthcare, Munich, Germany) after incubation of the membrane with ECL prime western blotting detection reagent (GE Healthcare).

### Histological analysis

Testes were fixed in 4% paraformaldehyde in PBS and embedded in Technovit 8100 resin (Heraeus-Kulzer, Wehrheim, Germany) according to the manufacturer’s instructions. Sections were cut at 5 μm and stained with periodic acid-Schiff (PAS) and then counterstained with Mayers hematoxylin solution (Wako, Japan).

### Immunohistochemistry

The spreading procedure of testicular cells was carried out as described [17]. In brief, hypotonic treated cells were spread and fixed on slides with 1% paraformaldehyde and 0.1% Triton X-100. Spread samples were blocked with 10% goat serum in PBS and then incubated with anti-SYCP3 (1:500) and anti-phospho-histone H2A.X (1:500) antibodies overnight at 4 °C in blocking solution. After incubation with Alexa Fluor 488 or Alexa Fluor 546-conjugated secondary antibody (1:250) at room temperature for 1 h, samples are counterstained with Hoechst33342, and mounted with Immu-Mount (Thermo Fisher Scientific).

## Results

### Expression of *Tesmin* isoforms

**Figure 1:**
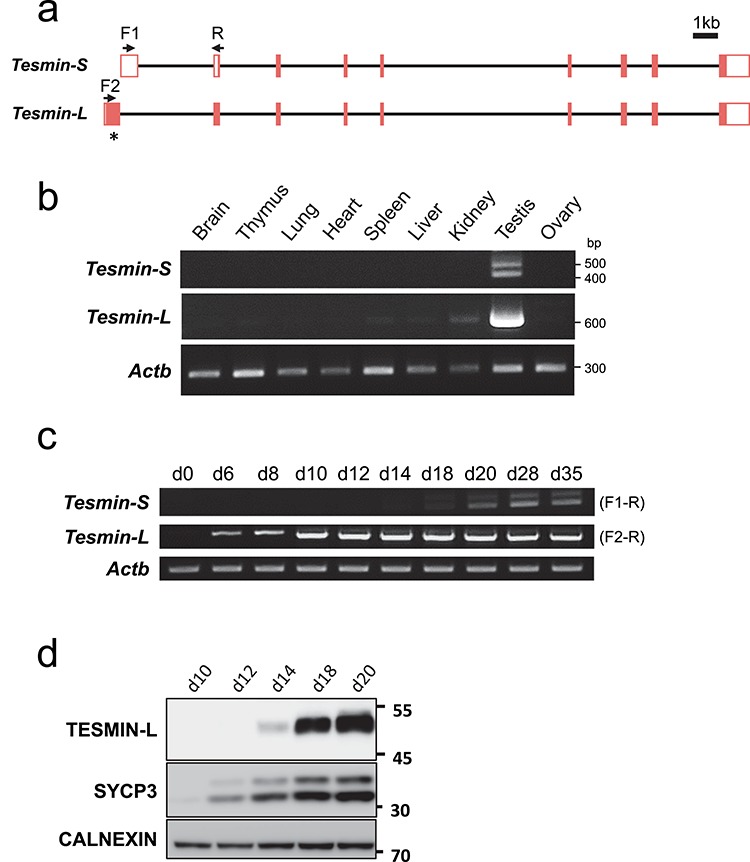
Expression profile of *Tesmin* isoforms. (a) Schematic representation of two splicing variants of mouse *Tesmin*. Filled box: protein-coding sequence, white box: untranslated sequences, arrows: primer binding sites. * shows the region that the antibody was raised against. (b) RT-PCR of *Tesmin* using cDNAs obtained from various tissues. *Actb* as control. (c) Expression of each variant analyzed with RT-PCR using testicular cDNA obtained from 0-, 6-, 8-, 10-, 12-, 14-, 18-, 20-, 28-, and 35-day-old mice. *Actb* as control. Primer sets used for each *Tesmin-S* and *Tesmin-L* are indicated at right, whose annealing sites are shown in Figure 1a. (d) TESMIN-L expression in spermatogenesis detected by western blotting. SYCP3 (a meiosis marker) and CALNEXIN (ubiquitously expressed marker) were used as controls.

*Tesmin* was originally identified as a gene encoding a testis-specific 30-kDa protein in mice [8] but later shown to have another splicing variant [9]. Because *Tesmin-L* and *Tesmin-S* transcripts utilize different starting exons but share common exons 2–9, we designed specific primers and performed RT-PCR with various adult tissues (Figure 1a). While the *Tesmin-S* signal was weak but specifically observed in testis, the *Tesmin-L* signal was strongly detected in testis with weak signals in spleen, liver, and kidney (Figure 1b). As shown in Figure 1b, we further detected two bands of *Tesmin-S* by RT-PCR. Sequencing analysis revealed that the upper band includes an additional 69 bp outside the coding region (Figure S1a). When we assessed the expression pattern of *Tesmin* variants during spermatogenesis (Figure 1c), *Tesmin-L* appears in a 6-day-old testis, which contains only spermatogonia and somatic cells, whereas *Tesmin-S* appears in a 18-day-old testis in which spermatocytes finish meiosis and haploid spermatids appear.

Next, to confirm the presence of TESMIN protein, we attempted to produce anti-TESMIN antibodies. One TESMIN-L specific antibody worked and detected a band near 50 kD, which is the expected size of TESMIN-L. Western blot analysis also detects TESMIN-L expression in a 14-day-old testis when differentiating germ cells enter the early pachytene stage of meiosis (Figure 1d). Since our anti-TESMIN-L antibody did not work for immunofluorescence in testis, we stained FLAG-tagged TESMIN-L and TESMIN-S transiently transfected into the Cos7 cell line with anti-FLAG antibody to know the localization of each variant (Figure S1b). Interestingly, TESMIN-L localized to the cytoplasm but TESMIN-S localized in close proximity to the nucleus, suggesting different roles between TESMIN-L and TESMIN-S in cells.

### CRISPR/Cas9-mediated generation of *Tesmin* KO mice

**Figure 2:**
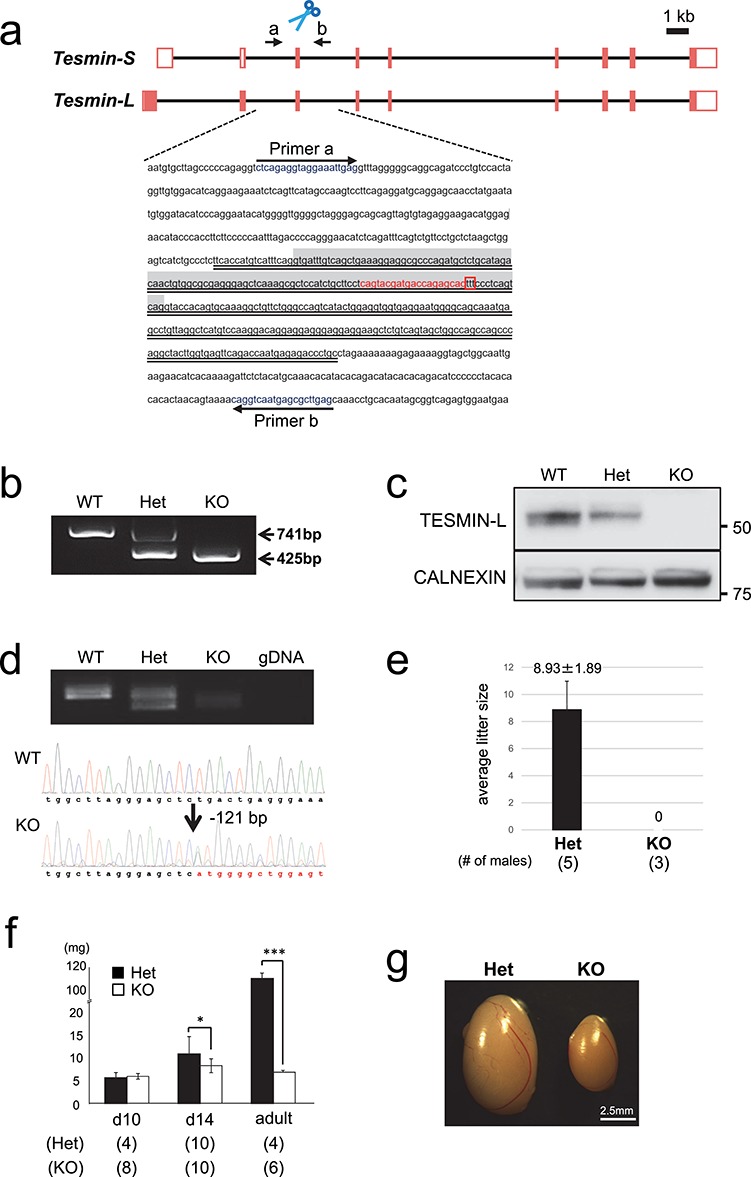
TESMIN deficiency leads to male infertility with spermatogenesis defects. (a) Targeting scheme of the *Tesmin* KO. *Tesmin* consists of nine exons. Exon 3, which is shared with both splicing variants, was targeted to generate the *Tesmin* KO mice. Sequence highlighted in gray indicates exon 3. Red characters and red box indicate the target of sgRNA and PAM sequence, respectively. Deleted region is underlined with a double line. Arrows indicate primers. (b) Genotyping by PCR. The 316-bp sequence was deleted in em1-mutant mice. (c) *Tesmin-L* expression in testis detected by western blotting. CALNEXIN (ubiquitously expressed marker) was used as a control. (d) Expression of *Tesmin* analyzed with RT-PCR using testicular cDNA obtained from *Tesmin* +/+, +/em1 and KO mice (top panel). Although a faint band was observed in *Tesmin* KO testis, the sequence of PCR products showed that *Tesmin* KO mouse had a 121-bp deletion that causes a frameshift (bottom panel). Red characters indicate the sequence after deletion. (e) Number of pups that were obtained by crossing *Tesmin* +/em1 (*n* = 5) and KO (*n* = 3) male mice with WT female partners. (f) Comparison of the testicular weight among *Tesmin* +/em1 and 10- and 14-day-old KO mice and adults. (g) Comparison of the gross morphology of the testis from *Tesmin* +/em1 and KO male adult mice (21 weeks old).

To analyze the physiological function of TESMIN proteins in vivo, we generated *Tesmin* KO mice using CRISPR/Cas9-mediated genome-editing in mouse zygotes. To disrupt both *Tesmin-L* and *Tesmin-S* genes, we designed a single guide RNA (sgRNA) against the common third exon (Figure 2a). We microinjected the sgRNA/Cas9 expressing plasmid into 105 zygotes and obtained eight newborn pups. By PCR amplification of the target region and subsequent sequence analysis, we confirmed three pups carrying mutations. While two of the three mice had indel mutations (+/i3 and +/i5d3), the third had a 316 bp-deletion (hereinafter, referred to as “em1” mutation) that includes the entire exon 3 (Figure 2b). Because exon 3 consists of 121 nucleotides, the lack of exon 3 results in a frameshift mutation in both *Tesmin-L* and *Tesmin-S* transcripts. We, therefore, bred this em1 mutant mouse line (B6D2-*Mtl5*<em1Osb>) to obtain *Tesmin* KO mice. *Tesmin* KO mice were born at the expected Mendelian ratio (+/+: 27, +/em1: 62, em1/em1: 29; *n* = 118), and no overt abnormality was observed in both females and males throughout their life. We confirmed the disappearance of TESMIN-L protein in the *Tesmin* KO mouse by western blotting (Figure 2c). Since an antibody against TESMIN-S was not available, we performed RT-PCR with *Tesmin-S* specific primers and confirmed the frameshift mutation (Figure 2d). These results indicate that both the TESMIN-L and TESMIN-S proteins are deleted in the *Tesmin* KO mice.

**Figure 3:**
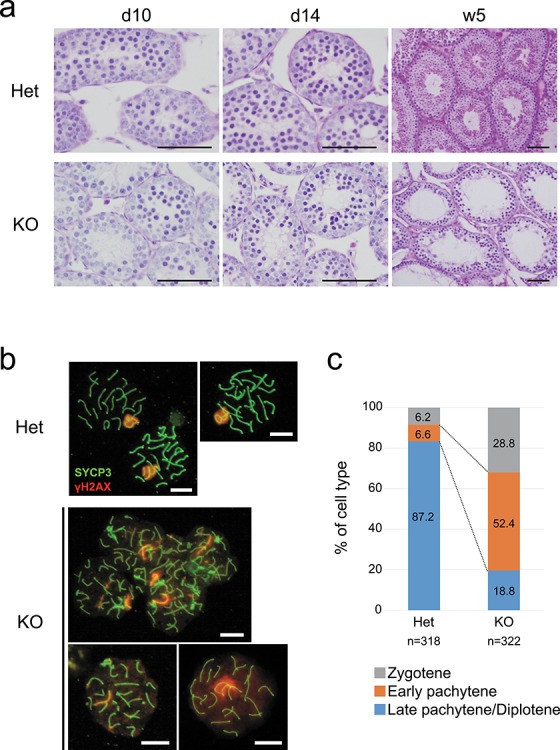
Spermatogenesis arrest occurred at meiosis. (a) PAS–hematoxylin staining of the testis from *Tesmin* +/em1 and 10-, 14-day-old, and 5-week-old KO mice . Scale bars: 50 μm. (b) Immunostaining of meiotic spreads from *Tesmin* +/em1 and KO spermatocytes for the synaptonemal complex component SYCP3 (green) to identify chromosome axes, and γH2AX (red) as a marker for DNA damage. Three spermatocytes from heterozygous are in late-pachytene stage. Top and bottom left spermatocytes of KO are categorized as early pachytene stage, and bottom right is categorized as late pachytene stage. Scale bars: 10 μm. (c) Percentage of spermatocytes from *Tesmin* +/em1 and KO male mice that are categorized as late zygotene, early pachytene and late pachytene by the feature of SYCP3 and γH2AX staining. Three mice (over 5 weeks old) are used for each genotype and more than 50 nuclei were observed from one mouse. The numbers in bar graph indicate the ratio (%) of each category. *P*-values of each categories are; zygotene: 0.0373, early pachytene: 0.0033, and late pachytene/diplotene: 0.0002, respectively. Welch *t* test was used for statistical analysis.

### Fertility of *Tesmin* KO male mice

To evaluate the effect of TESMIN deficiency on fecundity, *Tesmin* KO female (*n* = 10) and male (*n* = 3) mice were mated with WT males or females for 3 months, respectively. Whereas the *Tesmin* KO female mice were fertile (litter size of +/em1: 7.9 ± 2.0, number of litters *n* = 15, litter size of em1/em1: 7.1 ± 2.7, number of litters *n* = 15), the *Tesmin* KO male mice were infertile despite showing normal mating behavior (litter size of 0, Figure 2e). To define the causes of male infertility, we measured testicular weights in *Tesmin* +/em1 and KO mice. Although the testicular weight of *Tesmin* KO mice was comparable with that of *Tesmin* +/em1 10-day-old mice, a significant decrease appeared in testis weight in *Tesmin* 14-day-old KO mice and remained smaller throughout their life (Figure 2f and g). These results indicated that the lack of TESMIN impairs testis function.

### Meiotic defect in *Tesmin* KO male mice

To define the cause of the smaller testis in *Tesmin* KO mice, we observed testicular sections stained with hematoxylin and PAS, which detect nuclei and carbohydrate chains, respectively (Figure 3a). There were no significant differences between *Tesmin* KO and +/em1 testis cross sections in 10- and 14-day-old testes, respectively (Figure 3a). Sections from 5-week-old *Tesmin* KO testis showed apparent abnormalities compared with that of *Tesmin* +/em1 mice. Instead of three layers seen in control sections, only two layers of germ cells were observed at the periphery at 5 weeks in KO seminiferous tubules with a few cells beyond the meiotic stage (Figures 3a and  S2). These results indicated that spermatogenesis was predominately arrested at an early stage before the appearance of haploid spermatogenic cells. Due to an apparent defect in meiosis in the KO, we focused on the meiotic events and performed immunofluorescence staining of synaptonemal complex protein 3 (SYCP3) and γ-H2AX, a histone variant (Figure 3b). During meiotic prophase I, SYCP3 protein begins to assemble along each sister-chromatid pair at leptonema to form the axial elements, which appears as short filaments dispersed in the nucleus. Then, during zygotene, these filaments begin to associate in pairs to form thicker filaments, called the lateral elements of synaptonemal complex [18, 19]. Meanwhile, γ-H2AX, which is a marker of the response to DNA double-strand breaks, localizes throughout the entire nucleus during leptonema and early zygonema when programmed double-strand breaks are being generated by the Spo11 endonuclease [19]. Thereafter, it accumulates in the chromatin of the XY body from late zygotene until diplotene [18]. We prepared meiotic nuclear spreads from heterozygous and KO males and quantified zygotene, pachytene, and diplotene spermatocytes based on SYCP3 and γ-H2AX staining patterns according to published reports [20, 21]. In heterozygous, the major population (~80%) of selected spermatocytes showed a γ-H2AX foci in the XY body, which is the typical feature of late-pachytene to diplotene spermatocytes (Figure 3b and c). This distribution of spermatocytes among different meiotic stages was consistent with that of WT mice in a previous report [22]. However, in the majority of *Tesmin* KO spermatocytes, the staining pattern of γ-H2AX was not restricted to the XY body in fully synapsed chromosomes, which is found in zygotene and early-pachytene spermatocytes. These data suggest that meiosis was arrested before late-pachytene stage in the absence of *Tesmin*.

### Transgenic rescue of *Tesmin* KO mice

**Figure 4:**
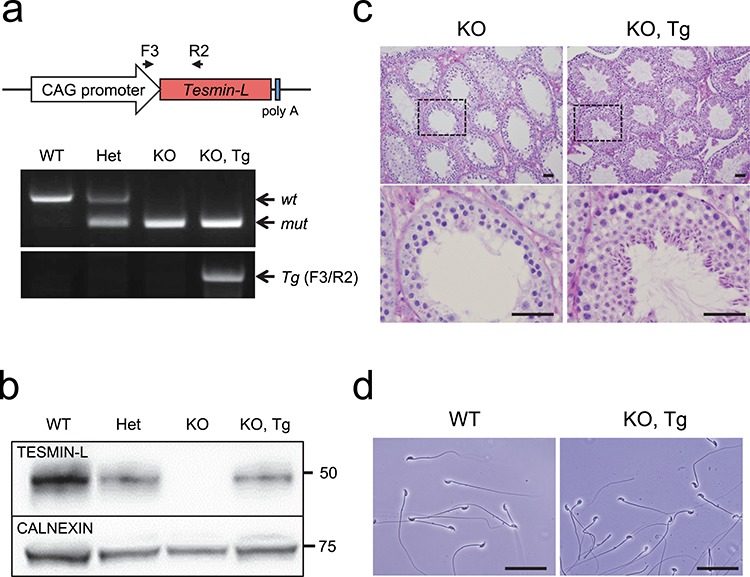
The sterile phenotype of *Tesmin* KO male mice was rescued by a *Tesmin-L* transgene. (a) Upper: schematic representation of the *Tesmin*-L transgene. Black arrows: primer annealing sites. Lower: PCR genotyping to check the *Tesmin* mutation and transgene. The *Tesmin* mutation was checked with primer set a/b described in Figure 2a and the *Tesmin*-L transgene was checked with primer set F3/R2. (b) TESMIN-L expression in *Tesmin* +/+, +/em1, KO and KO mice carrying the *Tesmin-L* transgene was detected by western blotting. CALNEXIN was used as a control. (c) PAS–hematoxylin staining of the testis from *Tesmin* KO and KO mice expressing the *Tesmin-L* transgene. Scale bars: 50 μm. (d) Cauda epididymal spermatozoa from *Tesmin* +/+ and KO mice. Scale bars: 50 μm.

To exclude the possibility that the infertility phenotype was caused by an off-target effect from CRISPR/Cas9 cleavage, or an aberrant genetic modification near the *Tesmin* locus, we performed a rescue experiment by generating transgenic mouse lines expressing *Tesmin-L* driven by the CAG promoter on a *Tesmin* KO background (Figure 4a). CAG promoter is a strong ubiquitous promoter frequently used to drive high levels of gene expression [13]. From pronuclear injection of 88 zygotes, we generated five founder lines. Western blotting analysis confirmed the expression of the transgene in these mice (Figure 4b). In *Tesmin* KO mice carrying the *Tesmin-L* transgene, the testicular weight (99.3 ± 9.8 mg) and spermatogenesis were comparable with the control *Tesmin* +/em1 mice (Figure 4c and d). In addition, the infertile phenotype was rescued (litter size of 7.8 ± 2.5, number of litters *n* = 21). These data that indicate infertility in *Tesmin* KO mice are due to the absence of TESMIN protein.

## Discussion

The study of MTs stretches back decades when they were first identified in the late 1950s [23]. MTs belong to the group of intracellular cysteine-rich, metal-binding proteins that have been found in bacteria, plants, invertebrates, and vertebrates [24]. Mammalian MTs are involved in diverse intracellular functions, with their role in detoxification of heavy metals and maintenance of essential metal ion homeostasis having been mostly investigated. However, TESMIN, which was identified as a MT-like protein (also known as *Mtl5*) and abundantly expressed in testes had not been studied well. In our experiments using KO mice, we demonstrated that TESMIN is an essential protein for meiotic progression in male germ cells.

Previous studies revealed two *Tesmin* splicing variants, named *Tesmin-S* and *Tesmin-L* in this paper [8, 9]. In this study, we confirmed that *Tesmin-S* and *Tesmin-L* encoding mRNAs that begin expression before and after meiosis, respectively and their localization in Cos7 cells were completely different. These results are consistent with the previous report, which claimed that TESMIN localizes in the cytoplasm of spermatocytes and then begins to localize on the nuclear membrane just under the acrosome vesicle of elongated spermatids [9]. It allows us to speculate that the splicing variants of *Tesmin* may have distinct roles in spermatogenesis. However, we demonstrated that the *Tesmin-L* transgene alone could rescue the meiotic defect and male fertility in *Tesmin* KO mice, suggesting that TESMIN-S is not essential for spermatogenesis.

A previous report showed that TESMIN-L localizes to the cytoplasm until the pachytene stage of meiosis then moves into the nucleus just before meiotic division [9, 11]. Our immunofluorescence staining of FLAG-TESMIN-L in Cos7 cells showed TESMIN-L localization in the cytoplasm, which is consistent with the first localization in testis. From immunofluorescence staining of meiotic markers SYCP3 and γ-H2AX in meiotic nuclear spreads, we found spermatogenesis arrests at the early pachytene stage in the majority of spermatocytes in *Tesmin* KO testis. The timing of the arrest in spermatogenesis corresponds to the change of *Tesmin-L* localization from the cytoplasm to nucleus, where TESMIN-L is thought to play an important role for meiosis in cytoplasm, and then moves into nucleus.

Previously, a similar phenotype, spermatogenesis arrest at the early pachytene stage with abnormal localization of γ-H2AX, has been reported in several mice lines deficient in proteins related to chromosome-structure during prophase I [25–27]. Based on these reports, TESMIN-L may be involved in such specific events in the nucleus at meiosis-I, but it could be indirect because of its cytoplasmic localization. Moreover, although a great majority of *Tesmin* KO spermatocytes are arrested at the early pachytene stage, a small fraction of cells complete meiosis and cease development at the metaphase/anaphase or haploid stage (Figure S2). To address these questions, more precise assessment of the arrested stages by immunofluorescence staining with other meiotic markers such as RAD51 and SYCP1 would be beneficial.

*Tesmin* was originally identified as an MT family-like gene because of its metal-binding, cysteine-rich domain, and early studies showed that metal ion-treated mice translocated TESMIN from the cytoplasm to the nucleus during meiosis [8, 11]. These results indicate that TESMIN responds to metal ions, like other MT genes; however, there are some differences between canonical MTs and TESMIN, as discussed previously [9]. TESMIN is ten times larger than other MT family proteins, and its cysteine composition is only 7.8% compared to 30% in other MT proteins (Figure S3). This suggests that the non-CXC regions of TESMIN might have different roles from other MTs. Furthermore, the metal-responsive element (MRE), a DNA sequence located upstream of MT family genes that are activated by the binding of the metal-responsive transcription factor (MTF-1), is not present upstream of *Tesmin* [9]. MTF-1 is known to be translocated from the cytoplasm into the nucleus upon heavy metal exposure or stress stimuli and bind to the MRE [28, 29]. The translocation of TESMIN in a similar manner to MTF-1 upon metal exposure suggests it may have a similar function during meiosis. Notably, with the absence of an MRE, *Tesmin* expression would be regulated by meiosis-specific transcription factors rather than in a metal-dependent manner.

The CXC in TESMIN are also present in LIN54, which is an essential cell cycle regulator. The CXC domains form a larger domain within LIN54 called the CXC-hinge-CXC (CHC) domain that is conserved in many LIN54 orthologs in various species, such as plants, invertebrates, and mammals [30–32]. Previous reports showed that LIN54 binds to the promoters of mitotic genes, such as cdc2 through the CHC domain and that LIN54 depletion through RNAi arrested the cell cycle [33, 34]. In addition, LIN54 has been assumed to change its subcellular localization in a cell-cycle-dependent manner [35]. Moreover, in *Drosophila,* mutation of a member of the CXC-domain family, *tombola* (*tomb*), which is expressed specifically in testis, fails to express *Cyclin B* and shows meiotic arrest in the male germline [31]. These reports allowed us to anticipate that TESMIN might have a similar function to LIN54 and *tomb* such as cell-cycle regulation in the nucleus at the end of meiosis during spermatogenesis.

Here, we demonstrated that TESMIN is essential for the completion of meiosis in male germ cells. Further studies on TESMIN will elucidate the mechanism of meiosis in male germ cells and shed light on the study of male infertility. We would expect that *Tesmin* KO mice would provide a clue to elucidate how TESMIN relates to meiosis in future research.

## Supplementary Material

S_I_ioaa002
