# Supplementary material for: Tesmin, Metallothionein-Like 5, is Required for Spermatogenesis in Mice
Source: Biol Reprod. 2020 Jan 9;102(4):975–83. doi: 10.1093/biolre/ioaa002 (PMC7124961; doi:10.1093/biolre/ioaa002)
Supplement: S_I_ioaa002 [file s_i_ioaa002.pdf]

Fig. S1 (Oji *et al.*)

a

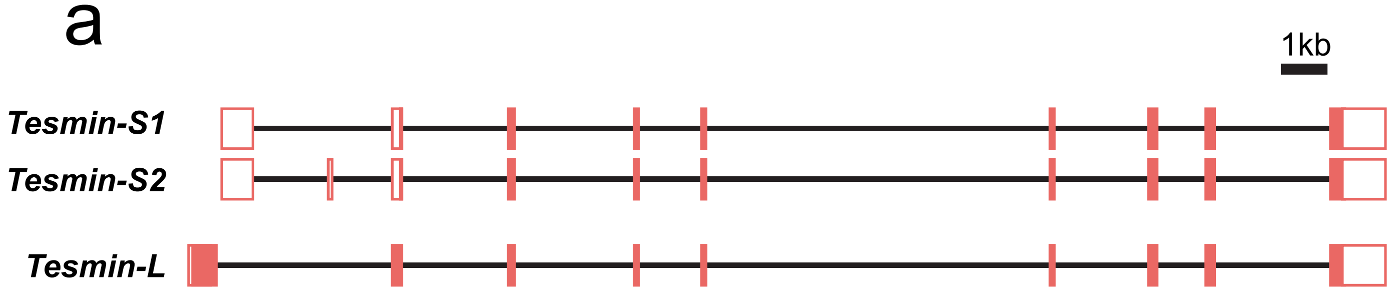

b

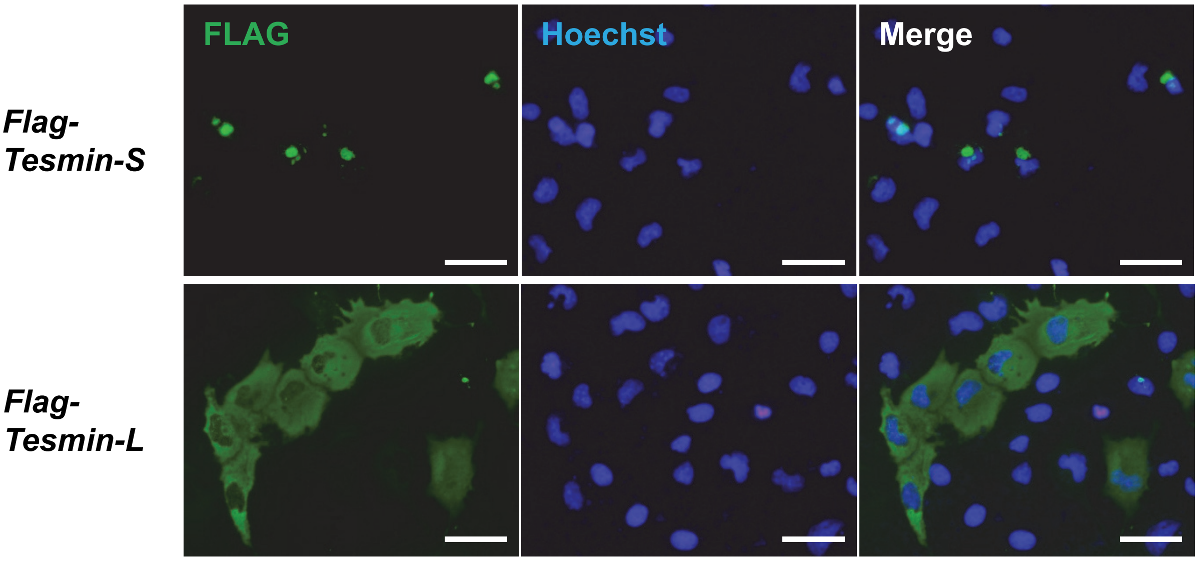

Fig. S2 (Oji *et al.*)

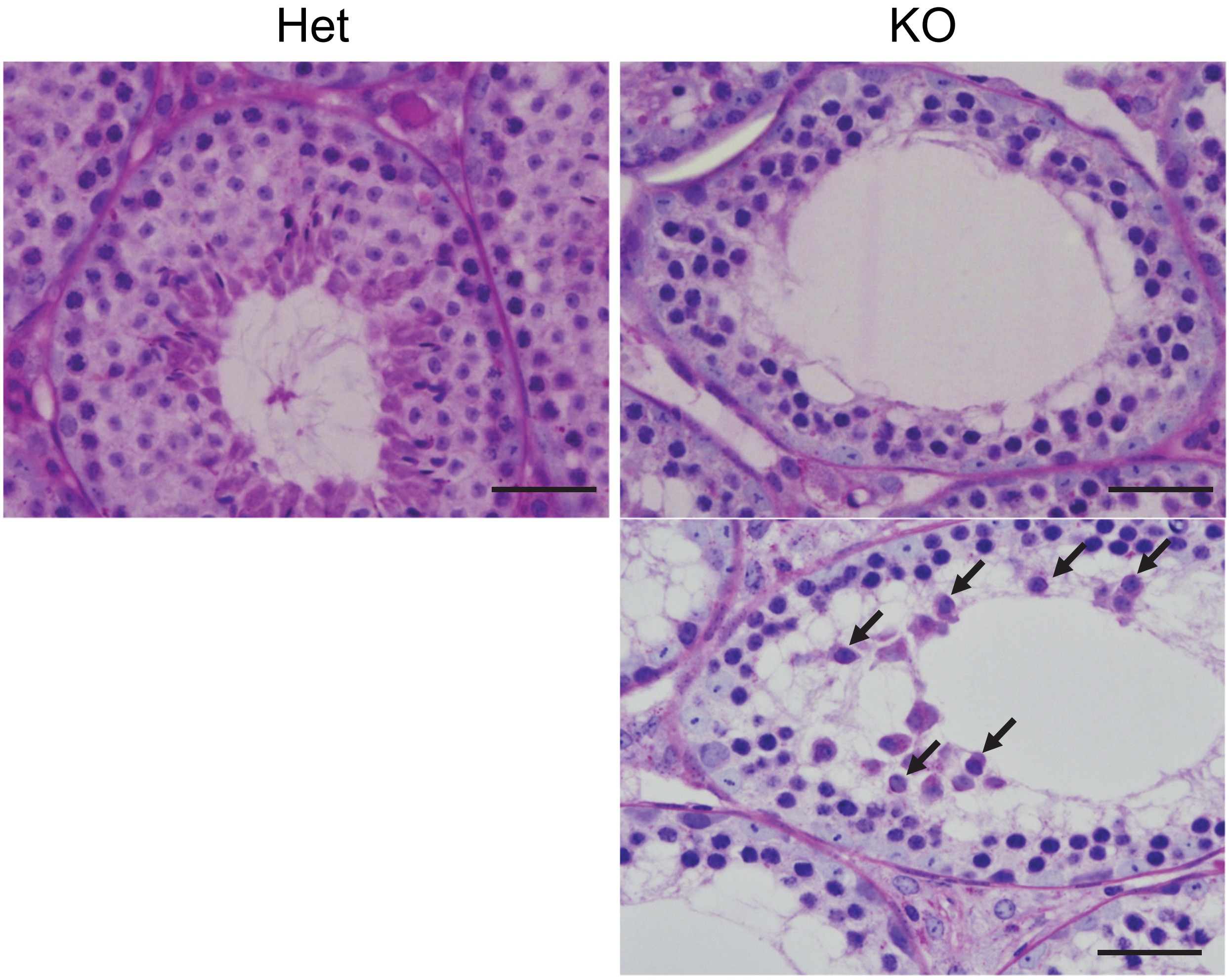

Fig. S3 (Oji *et al.*)

```
TESMIN-L 265 PALQGPPKITLSGYCDCFSSG-DFCNS-CSCNNLRHELERFKAIKACLDRNPEAFQPKMGKGRLGA 328
     MT1   1 ---------MDP-NCSCSTGGSCTCTSSCACK----------------------------------- 22
     MT2   1 ---------MDP-NCSCASDGSCSCAGACKCK----------------------------------- 22
     MT3   1 ---------MDPETCPCPTGGSCTCSDKCKCK----------------------------------- 23
     MT4   1 ---------MDPGECTCMSGGICICGDNCKCT----------------------------------- 23

TESMIN-L 329 AKLRHSKGCNCKRSGCLKNYCECYEAKIM-CSSICKCIACKNYEESPERKMLMSTPHYMEPGDFES 393
     MT1  23 -------NCKC--TSCKKSCCSCCPVGCSKCAQGCVCKG------AADKCTCCA*            61
     MT2  23 -------QCKC--TSCKKSCCSCCPVGCAKCSQGCICKE------ASDKCSCCA*            61
     MT3  24 -------GCKC--TNCKKSCCSCCPAGCEKCAKDCVCKGEEGAKAEAEKCSCCQ*            68
     MT4  24 -------TCSC--KTCRKSCCPCCPPGCAKCARGCICKG------GSDKCSCCP*            62
```
